# Confounding diagnosis of metastatic disease on FDG PET/CT due to cosmetic liquid silicone administration in breasts and gluteal regions with silicone migration to labia majora

**DOI:** 10.1016/j.radcr.2025.07.064

**Published:** 2025-08-16

**Authors:** Ya Ruth Huo B, Reece Tso, Lily Shen, Robert Mansberg

**Affiliations:** a Department Nuclear Medicine, Nepean Hospital, Derby St, Kingswood, NSW 2747, Australia; b Faculty of Medicine and Health, University of Sydney, City Rd, Darlington, NSW 2006, Australia

**Keywords:** Breast cancer, Silicone injections, False positive, FDG PET/CT, Silicone lymphadenitis

## Abstract

Cosmetic liquid silicone injections can result in silicone-induced inflammation, chronic mastitis, migration to distant locations and lymphadenitis which can result in false positive nodal and distant metastases. This study demonstrates a patient with multifocal FDG-avid right breast malignancy with FDG-avid bilateral axillary, mediastinal and abdominopelvic lymph nodes. She also had extensive FDG-avid foci in the bilateral breasts and subcutaneous gluteal regions secondary to cosmetic silicone administration. There was migration of injected silicone via the inguinal ligaments into her bilateral labia majora. In patients with extensive silicone injections, contrast-enhanced MRI, FDG-PET/CT and FES-PET/CT may assist in follow up for malignancy recurrence as the standard mammogram, ultrasound and CT follow-up demonstrates limited detection of interval changes.

## Introduction

Cosmetic liquid silicone injections can result in silicone-induced inflammation, chronic mastitis, migration to distant locations and lymphadenitis, which can result in false positive nodal and distant metastases. This report discusses a rare case of a patient with extensive FDG-avid silicone injections into the bilateral breasts with a multifocal FDG-avid right breast malignancy with FDG-avid bilateral axillary, mediastinal and abdominopelvic lymph nodes. The patient also had extensive silicone injections into the bilateral subcutaneous gluteal regions with migration of injected silicone through the bilateral inguinal ligaments into her labia majora.

## Case report

A 59-year-old woman presented with pain and inflammation of the right breast with history of bilateral breast and gluteal liquid silicone injections 15 years prior in the Philippines. On examination, she had a nodular right breast with skin changes in the right lower right breast and palpable right axillary lymph nodes. The differential diagnosis included silicone mastitis and breast malignancy.

**Fig. 1:**
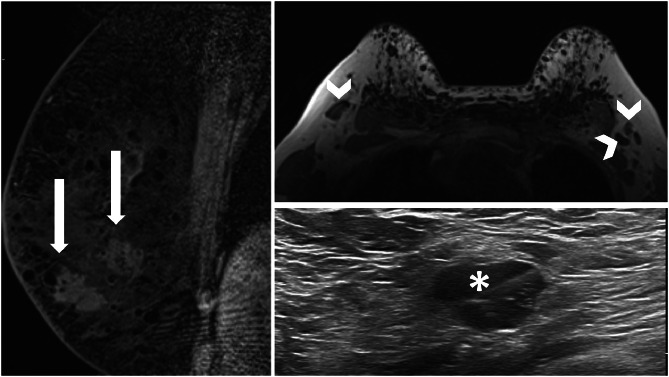
Left image – MRI right breast (postgadolinium T1 sagittal SPAIR sequence) demonstrated 2 hyperenhancing right lesions at 6 o'clock 5cm from the nipple (29mm lesion more superficial (left arrow) and 24mm deeper lesion) (right arrow), consistent with breast malignancies (MRI-guided biopsy proven). Top right image – MRI Breast (T1 axial sequence) demonstrated several enlarged (cortex > 3mm) bilateral axillary lymph nodes (arrowheads). Bottom right image – Ultrasound-guided biopsy of enlarged right axillary lymph node (asterisk) demonstrated metastatic breast malignancy.

Mammography and ultrasound were limited due to the extensive breast silicone injections reducing the visualization of underlying lesions. Breast MRI then demonstrated 2 enhancing right mass lesions at 6 o'clock 5cm from the nipple (29mm lesion more superficial and 24mm deeper lesion) and several enlarged bilateral axillary lymph nodes (Fig. 1). Ultrasound-guided right breast biopsy was unsuccessful due to shadowing from the silicone injections. MRI-guided biopsy of right superficial breast lesion demonstrated Grade 2 invasive carcinoma (no special type), estrogen- and progesterone- receptor positive, HER2 positive, Ki67 25%–40%. Ultrasound-guided biopsy of a right axillary lymph node demonstrated metastatic breast malignancy (Fig. 1).

**Fig. 2:**
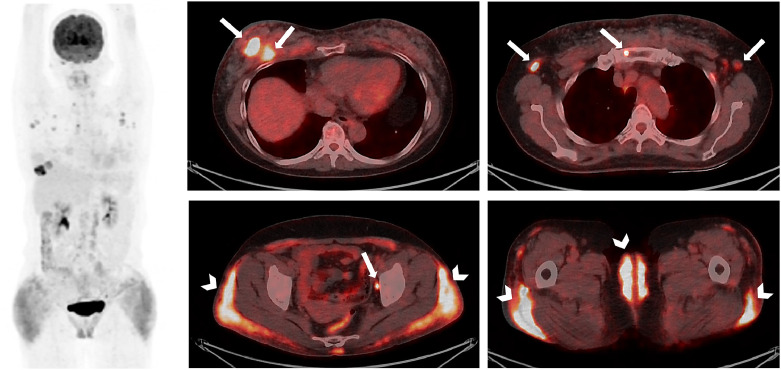
FDG-PET/CT demonstrated intense metabolic activity in 2 right breast malignancies (SUVmax 15.0, top left image, arrows) with metabolic activity in the bilateral axillary, mediastinal, para-aortic, and left pelvic lymph nodes (SUVmax 6.7) and sternal bony metastasis (SUVmax 7.6) (arrows). There was moderate metabolic activity in the bilateral subcutaneous gluteal foci, inguinal ligaments and bilateral labia majora (SUVmax 7.0), favored benign silicone-related inflammation (arrowheads, bottom right and left image).

FDG-PET/CT demonstrated 2 FDG-avid right breast malignancies (SUVmax 15.0) with FDG-avid bilateral axillary, mediastinal, para-aortic, and left pelvic lymph nodes (SUVmax 6.7) and bony metastases (SUVmax 7.6) (Fig. 2). This scan also demonstrated FDG-avid uptake in bilateral subcutaneous gluteal foci, inguinal ligaments and bilateral labia majora (SUVmax 7.0), favored due to prior silicone injection inflammation. FDG-uptake in the left axillary and left pelvic lymph nodes may be due to metastases or silicone lymphadenitis (not biopsied) (Fig. 2).

**Fig. 3:**
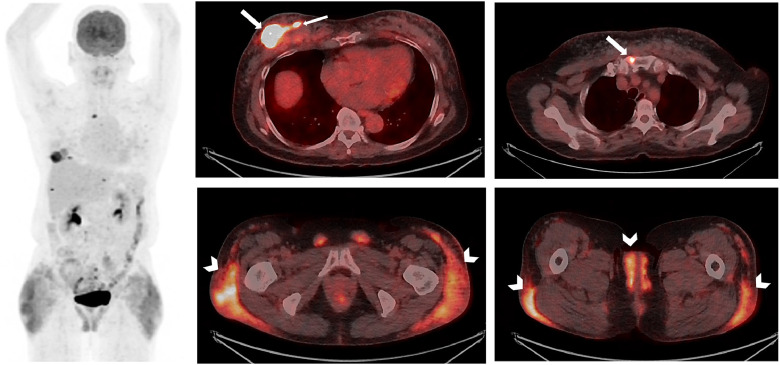
Following neoadjuvant chemotherapy, a progress FDG PET/CT was performed 6 months later. Intense metabolic activity in a new right medial breast lesion (SUVmax 8.2) (medial thin arrow) was demonstrated with interval partial reduction of metabolic activity in the remaining known 2 breast malignancies (SUVmax 15.0 to 10.1). There was reduced metabolic activity in the bilateral axillary, mediastinal, para-aortic, and left pelvic lymph nodes (SUVmax 6.7 to 2.1) as well as the sternal bony metastasis, favored to represent response to neoadjuvant chemotherapy (thicker arrows). Mild reduction in metabolic activity in the subcutaneous silicone injections (SUVmax 7.0 to 5.3), favoured variable silicone-related inflammation (arrowheads).

Following neoadjuvant chemotherapy, there was a new FDG-avid right breast lesion (SUVmax 8.2) with interval reduction of FDG-avidity in the remaining known breast malignancies (SUVmax 15.0 to 10.1) (Fig. 3). The new breast lesion was favoured to represent a synchronous breast malignancy with a less likely differential diagnosis of a siliconoma [1]. There was reduced FDG-avidity in the bilateral axillary, mediastinal, para-aortic, and left pelvic lymph nodes (SUVmax 6.7 to 2.1), favored to represent response to neoadjuvant chemotherapy. It is unclear whether the residual low grade FDG-avid lymph nodes were due to residual disease or silicone lymphadenitis [2]. A mild reduction in FDG-avidity in the subcutaneous silicone injections (SUVmax 7.0 to 5.3) was also noted (Fig. 3, arrowheads). FDG-avidity in bilateral inguinal ligaments and bilateral labia majora were favoured to be silicone migration from the gluteal injections [3], with migration from breast augmentation [4,5], direct silicone injections[6] and metastatic disease [6] considered less likely differential diagnoses.

## Discussion

This study highlights the diagnostic difficulty posed by cosmetic liquid silicone injections in the setting of initial staging of a breast malignancy with FDG PET/CT. FDG PET/CT is widely viewed as a sensitive modality for detecting malignancy, however it lacks specificity in differentiating inflammatory processes from malignant lesions [1,2]. The presence of FDG avid silicone granulomas significantly impairs diagnostic accuracy, often mimicking metastases or synchronous malignancies [1,7].

Silicone injections may induce granulomatous inflammation response, often leading to FDG uptake in local and distant tissues. Siliconomas or silicone induced lymphadenitis may often present with intense FDG avidity, making it difficult to distinguish from true malignancy [1]. In this case, FDG avid lymph nodes within the axilla, mediastinal, paraaortic, pelvic and labial regions raise concerns for disseminated disease versus benign inflammatory changes secondary to silicone migration. Being aware of these patterns can be helpful in raising suspicion of false positive findings.

False positive findings on PET/CT can have significant clinical consequences which can lead to unnecessary biopsies, treatments, and changes in staging. Ultimately, definitive differentiation requires tissue diagnosis to confirm a foreign body granuloma versus a malignancy. Cytology or histology classically reveals refractile silicone material with surrounding multinucleated foreign body giant cells which confirms siliconoma [8]. False positive PET due to siliconomas [9] demonstrate the importance of pathological correlation as highlighted in this study limiting the need for unnecessary oncological treatments.

Recent studies have demonstrated dual-energy CT and contrast-enhanced breast MRI with diffusion weighted imaging (DWI) and silicone sensitive sequences may help differentiate breast malignancy versus silicone granulomas [10]. A recent study demonstrated axial dual-energy CT with material decomposition and silicone-sensitive sequences can identify free silicone [10]. Silicone granulomas often demonstrate enhancement surrounding nonenhancing silicone deposits [10], delayed enhancement [11] and type 1 enhancement characteristics [12] (versus breast malignancy which often demonstrates morphological differences with a solid enhancement with rapid enhancement with washout; type 2 or 3 enhancement kinetics [12]). Unfortunately, MRI may not be as useful to assess distant lymphadenopathy versus nodal metastases.

The 18-F-fluroestradiol (FES) PET/CT imaging would likely be a useful alternative imaging modality to differentiate oestrogen-receptor breast cancer recurrence and silicone granulomas. Silicone granulomas are not known to have estrogen receptors and should not theoretically have uptake on FES-PET/CT [13]. A recent meta-analysis demonstrated 18-F-FES PET/CT imaging had better sensitivity than 18-F-FDG PET/CT in restaging oestrogen-positive breast malignancies (98% vs 81%, respectively) [13]. Future studies examining the usefulness of 18-F-FES PET/CT are warranted.

Overall, this study demonstrates the difficulty in assessing metastatic breast malignancy disease progression in the context of extensive silicone injections and the need for multidisciplinary imaging with MRI and PET/CT combined with pathology and clinical context. Silicone injections further confound findings of siliconomas [1], chronic mastitis [14], breast malignancy within silicone granuloma [15] and silicone migration to lymph, liver and spleen [16]. Awareness of these findings can help prevent misdiagnosis, incorrect staging, and treatment.

## Conclusion

This case highlights the difficulty differentiating residual malignancy or silicone-related inflammation in a patient with extensive FDG-avid silicone injections in bilateral breasts, gluteal, inguinal and labia majora regions with metastatic multifocal right breast malignancy with FDG-avid nodal and skeletal metastases. Moving forward, the patient’s follow-up plans must balance both diagnostic accuracy with radiation exposure. Contrast-enhanced breast MRI including diffusion weighted imaging (DWI) and silicone sensitive sequences may offer lower radiation surveillance in assessing breast lesions and silicone altered tissue. FES-PET/CT may also offer greater sensitivity than FDG-PET/CT. This case emphasizes the importance of personalized imaging strategies for patients with prior cosmetic silicone procedures which affect imaging. Whilst FDG/PET remains a valuable tool in detecting treatment response, limitations in determining FDG-avidity should be considered.

## Patient consent

The authors confirm that written, informed consent for publication of this case was obtained from the patient. This is retained with our records.
